# MiRNATIP: a SOM-based miRNA-target interactions predictor

**DOI:** 10.1186/s12859-016-1171-x

**Published:** 2016-09-22

**Authors:** Antonino Fiannaca, Massimo La Rosa, Laura La Paglia, Riccardo Rizzo, Alfonso Urso

**Affiliations:** National Research Council of Italy, ICAR-CNR, via Ugo La Malfa 153, Palermo, 90146 Italy

**Keywords:** miRNA, SOM, mRNA, Target prediction, miRNA-mRNA interactions

## Abstract

**Background:**

MicroRNAs (miRNAs) are small non-coding RNA sequences with regulatory functions to post-transcriptional level for several biological processes, such as cell disease progression and metastasis. MiRNAs interact with target messenger RNA (mRNA) genes by base pairing. Experimental identification of miRNA target is one of the major challenges in cancer biology because miRNAs can act as tumour suppressors or oncogenes by targeting different type of targets. The use of machine learning methods for the prediction of the target genes is considered a valid support to investigate miRNA functions and to guide related wet-lab experiments. In this paper we propose the miRNA Target Interaction Predictor (miRNATIP) algorithm, a Self-Organizing Map (SOM) based method for the miRNA target prediction. SOM is trained with the seed region of the miRNA sequences and then the mRNA sequences are projected into the SOM lattice in order to find putative interactions with miRNAs. These interactions will be filtered considering the remaining part of the miRNA sequences and estimating the free-energy necessary for duplex stability.

**Results:**

We tested the proposed method by predicting the miRNA target interactions of both the Homo sapiens and the Caenorhbditis elegans species; then, taking into account validated target (positive) and non-target (negative) interactions, we compared our results with other target predictors, namely miRanda, PITA, PicTar, mirSOM, TargetScan and DIANA-microT, in terms of the most used statistical measures. We demonstrate that our method produces the greatest number of predictions with respect to the other ones, exhibiting good results for both species, reaching the for example the highest percentage of sensitivity of 31 and 30.5 *%*, respectively for Homo sapiens and for *C. elegans*. All the predicted interaction are freely available at the following url: http://tblab.pa.icar.cnr.it/public/miRNATIP/.

**Conclusions:**

Results state miRNATIP outperforms or is comparable to the other six state-of-the-art methods, in terms of validated target and non-target interactions, respectively.

## Background

MicroRNAs (miRNAs) are small non-coding single stranded RNA molecule, 22–25 nucleotides (nt) long, found in many organisms (plants, animals, and some viruses) [1]. MiRNAs are important players in gene regulation. The most important step in their regulatory function is the targeting of RNA messengers (mRNAs). MiRNAs, in fact, are responsible for degradation or repression of mRNAs at post-transcriptional level, when their sequences bind with partially complementary sites. This way, they play a crucial role in the cell differentiation and proliferation, apoptosis, and many other physiological and pathological processes [1].

Expression patterns of miRNAs are highly related to specific external stimuli, developmental stage or tissue. For example, in cancer disease the expression levels of miRNAs are known to change considerably [2].

Many recent works proved a different behaviour of cellular actors mediated by a differential expression of miRNAs that are cell condition or tissue/specific [3]. In the pathology of cancer this is relevant as they can act as tumour suppressors or oncogenes by targeting different type of targets, leading respectively to decrease or accelerate the tumorous processes. Thus, analysing the miRNA-mRNA interaction would mean to better understand the molecular mechanism of the pathological condition compared to the normal cell behaviour, through the main actors that are proteins, and moreover to hypothesize new therapeutic strategies of intervention to stop the malignant processes [4, 5].

MiRNAs were first identified in 1993 [6] via classical genetic techniques in Caenorhabditis Elegans (Nematoda; Rhabditidae).

It is just over the last decade that thousands of miRNAs have been discovered in all kinds of taxa and their regulations in cancer have been analysed [7–9]. Unfortunately, most of these studies were focused only on a specific subset of miRNAs, or a limited group of patients. The role of miRNAs was also demonstrated in the early stages of the disease progression and metastasis. In fact, several experimental evidences showed miRNAs are involved in the regulation of those biological processes, leading to the acquisition of metastatic potential, including adhesion, invasion, migration, epithelial-mesenchymal transition and angiogenesis [10, 11].

MiRNAs interact with their mRNA targets especially by base pairing in the 3’-untranslated regions (3’UTR) of mRNA sequences. In living species, near perfect base pairing is required between the so called miRNA seed, i.e. the first 8 nt in the 5’ miRNA sequences, and a target site in the 3’UTR mRNA sequences. In plants, the whole miRNA sequences usually have near-perfect pairing with their mRNA targets, which induces gene repression through cleavage of the target transcripts. In contrast, with few exceptions, in animals, the base pairing between the whole miRNA sequences and their mRNA targets is imperfect. However, some authors have identified three main rules for miRNA-target base pairing by experimental and in silico analysis [12]:

1. Perfect and contiguous base pairing of miRNA seeds, made of nucleotides 2 to 8 in 5’ miRNA, which nucleates the miRNA-mRNA association. In general, conditions as mismatches and bulges in the seed region should be avoided because it greatly affect on repression.
2. There must be enough complementarity to the miRNA 3’ half in order to stabilize the interaction. In this region bulges and mismatches are generally allowed.
3. The central region of the miRNA-mRNA duplex should have bulges or mismatches, in order to preclude the endonucleolytic cleavage of mRNA.

Because experimental identification of miRNA targets is a difficult work, the aid of computational tools for target predictions is a valuable instrument to investigate miRNA functions and to guide related wet-lab experiments. Usually two research problems involving miRNA are addressed with computational methods, i.e. miRNA genes detection and miRNA targets prediction. The former consists in the identification of those regions in the genome that produces the miRNAs; the latter searches for the mRNAs that could interact with the miRNAs. Machine learning methods have improved the performance of both miRNA gene detection and target prediction [13–15]. These approaches typically make use of sequence data (e.g. of short 6–8 nt miRNA binding motifs), secondary structure (e.g. stem-loops using thermodynamic modelling) and evolutionary conservation to identify putative candidates, using algorithms such as Hidden Markov Models (HMM) [15], Random Forest classifiers [14] or Support Vector Machines (SVM) [13].

In this work, we present miRNATIP (miRNA Target Interaction Predictor), a method for miRNA target predictions based on Self-Organizing Maps (SOM). SOM networks [16] are artificial neural networks widely used to categorize large high-dimensional datasets by mapping the data into a smaller dimensional space, typically into a two-dimensional lattice of interconnected neurons. Each neuron of a SOM represents a reference model, corresponding to a local domain in the input space [17]. By using a competitive learning, rather than an error-correction learning like the back-propagation with gradient descent adopted by other artificial neural network algorithms, the SOM algorithm tries to reproduce the self-organizing mechanism that creates the somatosensory in some areas of the brain. In this sense the SOM algorithm can be defined as an artificial neural network, like many other algorithms inspired by neurons in the brain. The SOM is more than a clustering algorithm because it gives a visualisation of the distribution of the patterns in the input space. When the input patterns are projected on the map the clusters can be visualised, and the map can be divided into areas where the input patterns share some feature values. In our work, the SOM algorithm is able to cluster together the miRNA seeds and, consequently, to project on the trained lattice the 3’UTR mRNA sequences in order to find a preliminary list of putative targets. This list will be filtered out considering the remaining parts of both miRNA and mRNA sequences and finally it will be shortened using a threshold over the free-energy, whose values provides hints about the thermodynamic stability of the miRNA-mRNA duplex [18]. In bioinformatics, SOM has been previously applied to issues like clustering of protein sequences [19] and molecular compounds [20], gene finding [21], and identification of transcription factor binding sites [22].

The paper has the following structure: the next Section describes some related works about miRNA-target predictors; “Methods” section reports in details our proposed algorithm and the datasets used in our experiments; the basic SOM algorithm and some details of the other algorithms used for comparison; “Results and discussion” section reports both the methodology to tune the the parameters of miRNATIP algorithm and the the experimental prediction results compared with other six state-of-the-art miRNA-target prediction tools. Finally, some conclusion as well as our future work are reported in “Conclusion and future work” section.

## Related works

All known miRNA-targets are mainly based on experimentally validated miRNA-mRNA interactions [23, 24]. However, they represent only a very small part of all existing interactions. For this reason, in recent years several miRNA-target predictors have been developed. The available algorithms were recently reviewed in [18, 25, 26] focusing on their bioinformatics, mathematical and statistical features. In the following it will be discussed some of these in more detail.

The miRanda algorithm [27] searches for target sites on the 3’UTR regions of mRNAs. It considers both the binding energy for the duplex stability and the conservation of the target site among different species. Those miRNAs having multiple binding sites within 3’UTR are highly scored.

PicTar [28] identifies a list of putative targets searching for almost fully complementarity sites between miRNAs and 3’UTR mRNAs. The free energy between the binding sites is then computed and finally the results are ranked by means of a score obtained using an HMM, and miRNAs having multiple binding sites are highly scored. In order to refine the identified targets, PicTar looks for the target site conservation among eight vertebrate species.

TargetScan [29] algorithm is based on the identification of full complementary zones between the miRNA seed (nucleotide 2 to 7) and 3’UTR mRNA. Starting from those sites, TargetScan searches for larger interactions, ranking the results in three groups according to the length of the matches. In particular, the presence of an adenine in the first position of the target site is highly scored because of its evolutionary conservation.

DIANA-microT [30] scans putative target sites by means of a 38 nt-long sliding window moved over the 3’UTR region of mRNA. At each shift, the minimum free energy between the miRNA-mRNA binding sites is computed and then it is compared with the energy related to the supposed full (100 %) miRNA-mRNA complementarity. miRNA seed matches of 7, 8 and 9 nt are allowed, as well as 6 nt-long matches if there are further complementary sites in the remaining region of miRNA.

PITA [31] algorithm consists of two steps. In the first one, it looks for putative target sites considering near perfect complementarity between miRNA seed and 3’UTR mRNA. In the second step, PITA takes into account the actual accessibility of the target site, related to the transcript secondary structure, by combining the free energy of the miRNA-mRNA bound and the energy needed to unfold the mRNA and make it accessible.

RNAhybrid [32] searches for miRNA target sites considering the hybridization sites having the most advantageous energy content. Hybridization is a technique that measures the degree of genetic similarity between groups of DNA/RNA sequences [33] and it is usually used to determine the genetic distance between two organisms. RNAhybrid looks for targets in the 3’UTR mRNA.

MirSOM [34] is, at the best of our knowledge, the only other miRNA target prediction tool implementing a SOM. It takes *C. elegans* 3’UTR sequences and cluster them using a SOM in order to identify potential miRNA target sites. The SOM is built upon is a 32×32 grid and it is trained considering all the overlapping 22 nt-long fragments extracted from the 3’UTR mRNA sequences. At the end of the learning phase, MirSOM produces clusters of putative target sites. Then miRNA sequences are assigned to a mRNA cluster if they have a perfect match between their 7 nt-long seed and the last 8 nucleotides of the sequences belonging to that cluster. Because the SOM clusters together not only identical but also similar sequences, it is possible to identify miRNA-mRNA interaction having near perfect seed matching. At this point, each cluster contains a list of putative miRNA targets. Those list are filtered lefting out those miRNA-mRNA couples whose free energy is below a certain threshold.

MirSOM performed well against most other tools with high sensitivity and vastly improved specificity. Unfortunately, it currently supports only *C. elegans* data. The mirSOM interface allows the user to enter an miRNA and the predicted mRNAs are returned as output. mirSOM can be accessed from https://bioinformatics.uef.fi/mirsom/.

## Methods

In this Section, it is described the representation adopted for the miRNA and mRNA sequences; then it is presented the four-steps algorithm for the identification of miRNA targets and finally all the datasets used for our experiments are introduced.

### Genomic sequence representation

One of the major challenges in bioinformatics is finding the best representation of the DNA/RNA sequences. In our approach, similarly to [34], we represented RNA (miRNA and mRNA) sequences by means of a numerical encoding derived from the position weight matrices (PWMs) [35]. A PWM is a commonly used representation model in biological sequence analysis, obtained by computing the frequency of each specific base (A, C, G and T or U) at each nucleotide position in the sequence. The PWM model has been successfully applied to many problems in DNA and protein sequence analysis, for example in the identification of functional sequence elements [36].

In particular, within our method, each RNA sequence is represented with a PMW of 4×*k* elements, where 4 are the nucleotide symbols and *k* is the length of the sequence. Each column *j* has a fixed value according to the corresponding nucleotide in the *j*-th position, with 1≤*j*≤*k*. Numeral encoding for each nucleotide was the following: A = [1000]^*t*^, C = [0100]^*t*^, G = [0010]^*t*^, T/U = [0001]^*t*^

To measure the dissimilarity between two PWMs we considered, among the others [37], the normalised Euclidean Distance defined as:

1 $$ D(a,b) = \frac{1}{\sqrt{2k}} \cdot \sum_{j=1}^{k} \sqrt{\sum_{b\in \left\{A,C,G,T\right\}} \left(P_{j,b}^{1} - P_{j,b}^{2}\right)^{2}}   $$

where *P*^1^ and *P*^2^ are two PWMs, *k* is the length of the sequences and *P*_*j*,*b*_ is the values in column *j* with base *b*. This distance ranges from 0 (perfect identity) to 1 (complete dissimilarity).

### MiRNATIP pipeline

**Fig. 1:**
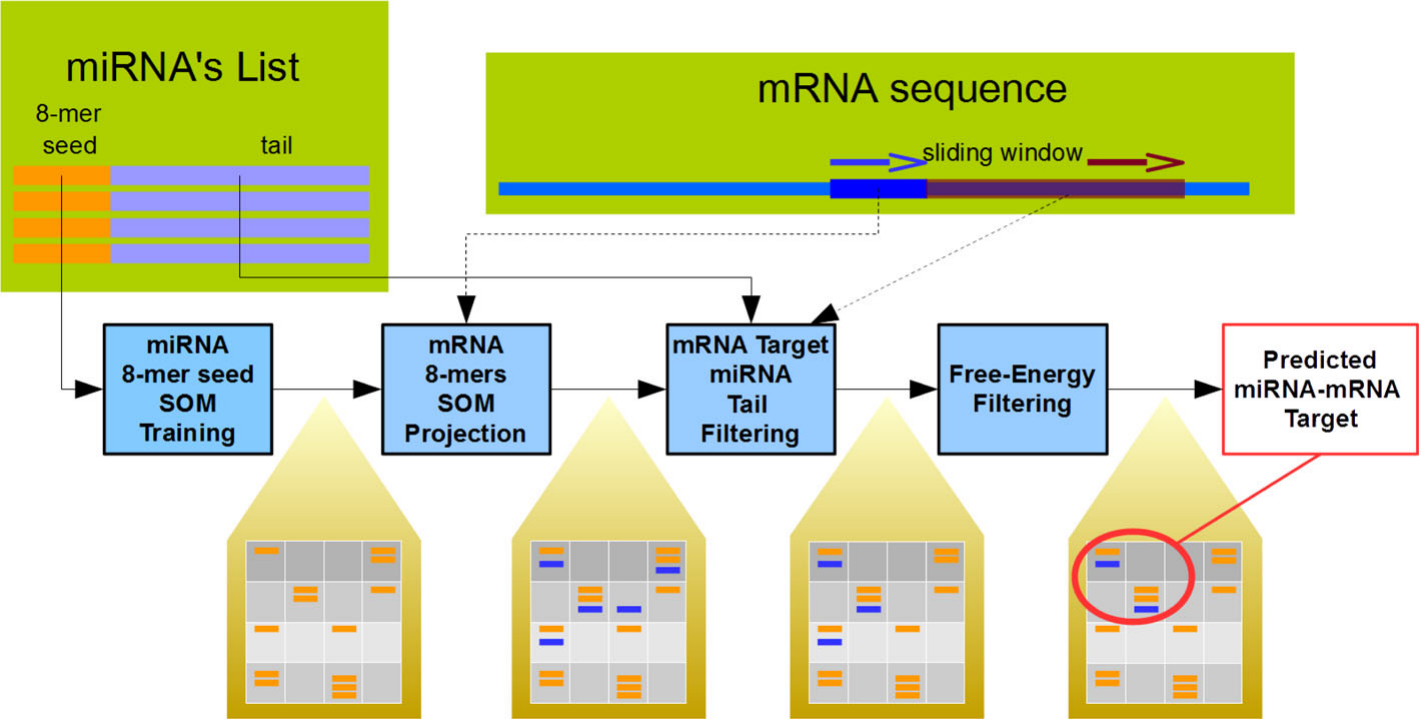
The proposed miRNA target prediction method. It is composed of four steps, each one represented by a light-blue coloured box. We described SOM training in section “SOM training”, SOM projection in section “SOM projection”, miRNA tail filtering in section “Tail filtering” and the free-energy filtering in section “Free-energy filtering”

The miRNATIP algorithm is composed of four main steps. Figure 1 shows the whole pipeline used in this work and it will be explained in detail in the following subsections. Steps 1 to 3 have been implemented using the Java programming language, so that miRNATIP is platform-independent.

#### SOM training

In the first step, a set of miRNAs seeds, fixed at a length of 8 nt, is used for the training of a SOM. More details on the SOM training algorithm can be found in [16]. We considered only the 8-mer miRNA seeds, because it has been demonstrated that the seed is mainly responsible of the miRNA target binding (cfr. Background). Each neuron is represented by a PWM of size 4×8 that are first initialised using random values. In this work, we used the batch method for the training of the SOM [38]. Furthermore, the neurons are arranged in a rectangular lattice, in which each neuron is connected to its four neighbours, except for those at the edge of the grid. To locate the best matching unit (bmu), it is calculated the distance between the input vector and the weights of each neuron according to Eq. 1. The result of this step is a set of clusters composed of the 8-mer seeds belonging to each miRNA.

#### SOM projection

The second step consists in the projection of a mRNA sequence over the trained SOM. For this reason, we extracted all the 8-length mRNA fragments through a 8-mer sliding window with step = 1. This way, we obtained a set of 4×8 PWMs that can be projected over the trained SOM. The result of this step is, for each neural unit (cluster), a list of couples (miRNA_seed, mRNA fragment). Each cluster can be considered as a preliminary set of predicted miRNA-mRNA interactions.

#### Tail filtering

In this step, we filtered those preliminary interactions considering the remaining part of the miRNA sequences, called miRNA_tail. For each couple (miRNA_seed, mRNA fragment), we considered respectively the miRNA_tail and the mRNA sequence of the same length of miRNA_tail, next to the projected mRNA fragment. Then we computed a dissimilarity measure based on normalised euclidean distance (Eq. 1) between the PWM representation of those two sequences and, according to the the rule no. 2 of the “Background” Section, we retained only the couples of miRNA-mRNA interactions whose distance is below a certain threshold. As reported in the third rule of the “Background” section, in order to take into account also the presence of possible bulge loops between the 8-mer seed and the tail of the miRNA, we considered an offset of few nucleotides (2–3), causing a shift of the mRNA fragment corresponding to the miRNA_tail.

#### Free-energy filtering

In the last step, we applied a further filtering process to the couples list, based on the minimum free-energy required to form the miRNA-mRNA duplex. For this purpose we used the IntaRNA tool [39, 40]. IntaRNA is able to calculate a free-energy value (given in kcal/mol) from a couple of genomic sequences, considering two different contributions: (1) the free-energy required to unfold the interaction sites both in miRNA and mRNA and (2) the hybridization free-energy between interacting nucleotides of genome sequences. The sum of these two contributions represents the final free-energy score. In our method, we introduced a threshold on this free-energy score: in this way, the putative interactions that obtain a free-energy score over the threshold are removed from the final miRNA-mRNA interaction list.

### Datasets

In our study, we focused on two species: *C. elegans* (cel) and human (Homo sapiens - hsa). Human species of course has been chosen for the importance that miRNA target interactions have with regards to regulatory functions involving many diseases, such as cancer. Moreover, we considered *C. elegans* because Nematodes have been studied in a wide range of fields, and they are organisms that allow to help to understand the molecular biology of humans and animals. They are easy to study thanks to their intrinsic features and handiness in cultivation and manipulation. miRNA mature sequences, both for cel and hsa, have been downloaded from miRBase [41] (release 21, update in June 2014), the most comprehensive online database of published validated miRNA sequences and annotation. We obtained 434 and 2588 miRNA sequences for cel and hsa, respectively. As for cel, the validated 3’UTR mRNA sequences are available on WormBase [42] (release WBcel 235, update in April 2013), an online genome database of the nematode model organism *C. elegans*, and they have been downloaded through the BioMart [43] online service. As for the hsa, the validated 3’UTR mRNA sequences were downloaded from Ensembl repository (release 80) [44]. We obtained a total of 30939 and 154666 3’UTR mRNA sequences for cel and hsa, respectively.

Experimentally validated miRNA-mRNA interactions, representing positive examples, were downloaded from mirTarBase [45], a repository of manually verified miRNA target interactions for the most studied species, including cel and hsa. We collected 3209 and 39111 positive validated interactions for cel and hsa, respectively. Finally we considered a set of validated non-target interactions, representing negative examples: for cel we had 16 non-target interactions; for hsa we collected 123 negative validated interactions. Thirteen out of 16 negative interactions for cel have been provided by [34], the remaining 3 and all the negative interactions for hsa have been found in Tarbase (release 7.0) [46], that is a publicly available database containing both miRNA-mRNA target and non-target interactions.

## Results and discussion

In this Section we describe how we selected the best parameter configuration for miRNATIP algorithm and then we analyse the prediction results against other six state-of-the-art miRNA-target interaction prediction tools.

### MiRNATIP configuration

During the SOM training step (see Fig. 1), in order to obtain the best parameters for the network learning, we performed several tests at varying of network size and learning rate *α* [16]. The quality of the trained map was measured by means of two evaluation criteria: resolution and topology preservation. These two measures are calculated respectively with the average quantization error (QE) and the topographic error (TE), as defined in [47]. We chose the configuration of SOM parameters that minimize both the QE and TE, according to the Eq. 2, where *c* is the configuration we adopted, *i* is a triple of SOM parameters (size, *α*_*max*_ and *α*_*min*_), $QE^{\prime }_{i}$ and $TE^{\prime }_{i}$ are respectively the normalized value of *QE* and *TE* for the triple *i*.

2 $$ c = \underset{i}{\text{argmin}} \left(mean \left(QE'_{i}, TE'_{i} \right) \right)   $$

**Fig. 2:**
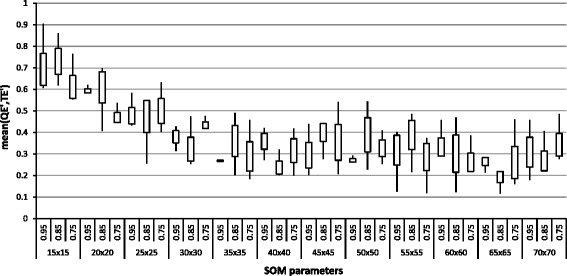
Measure of SOM training for hsa species at varying of SOM parameters. The box-plot reports the distribution of the mean between QE’ and TE’ (as defined in section “MiRNATIP configuration”), at varying of SOM parameters, i.e. map size (from 30×30 to 70×70) and *α*_*max*_ (from 0.75 to 0.95). Values of *α*_*min*_ (from 0.001 to 0.1) are omitted from the graph for the clarity of image. According to Eq. 2, the best configuration for hsa species is map size =65×65, *α*_*max*_=0.85 and *α*_*min*_=0.1

Figure 2 reports a box-plot that shows a trend of performed test at varying of *i* for the homo sapiens. At the end of the training phase, we obtained a configuration *c* with the following values: map size= 65×65, initial *α*= 0.85, final *α*= 0.1. The same configuration process has been computed for the *C. elegans* species. After this phase, the projection of mRNA fragments over the SOM lattice was performed.

As regards the third step of the proposed method, i.e. the tail filtering, we used a threshold of 0.7 over the euclidean distance (Eq. 1), that allow to preserve at least the 30 *%* of miRNA-mRNA match in the tail region. In addition, to simulate the presence of a bulge between the seed and the tail of the miRNA binding site, we set the offset =3, i.e. we supposed a bulge could contain at most three nucleotides. Finally, for establishing the minimum free-energy score, we performed different measurements of the obtained predictions and estimated the optimal threshold for cel and hsa, respectively equal to –6 and –7 kcal/mol.

All the configuration parameters used in this work are reported in Table 1.

**Table 1:** Parameters used for cel and hsa miRNA-target predictions

MiRNATIP parameters						
Species	SOM training			Tail filtering		Free-energy filtering
	Map size	*α*_*max*_	*α*_*min*_	offset	distance threshold	score threshold
*C. elegans*	30 ×30	0.95	0.1	3	0.7	–6 kcal/mol
Homo sapiens	65 ×65	0.85	0.1	3	0.7	–7 kcal/mol

The first column reports the species, the next three columns contain parameters for SOM training (section “SOM training”). Forth and fifth columns report parameters for miRNA tail filtering process (section “Tail filtering”). Finally, the last column shows the free-energy threshold score (section “Free-energy filtering”)

### Prediction results

MiRNATIP has been run using the datasets presented in “Datasets” section. Prediction results have been compared with those provided by other miRNA target predictors: PITA [31], MiRanda [27], MirSOM [34], PicTar [28], DIANA-microT [30], TargetScan [29]. These predictors have been considered for comparison because they allow to directly download the whole set of miRNA target predictions. MirSOM only provided prediction for cel species; as for TargetScan, the predictions were extracted by means of the miRDIP portal [48]. In order to obtain the most reliable and comparable results as possible, we filtered out the predictions of the other algorithms according to the following criteria. PITA and MiRanda predictions have been chosen considering the same free-energy thresholds we adopted for miRNATIP algorithm (–6.0 kcal/mol for cel and –7.0 kcal/mol for hsa). DIANA-microT predictions have been selected according to the default scores suggested by the authors (0.6 for cel and 0.7 for hsa). Finally for TargetScan we considered the conserved predicted targets, representing the most reliable interactions. In order to evaluate the correctness of a predicted miRNA-mRNA interaction, for each predictor we considered only the set of interactions that involve at least one miRNA/mRNA belonging to the datasets of experimentally validated miRNA-mRNA interactions. Prediction scores have been computed considering the following statistical measures [49]:

3 $$ Accuracy\ (ACC) = \frac{TP+TN}{P+N}   $$

4 $$  Precision\ or\ positive\ predictive\ value\ (PPV) = \frac{TP}{TP+FP}  $$

5 $$ Sensitivity\ or\ true\ positive\ rate\ (TPR) = \frac{TP}{TP+FN}  $$

6 $$ Specificity\ or\ true\ negative\ rate\ (TNR) = \frac{TN}{FP+TN}  $$

7 $$ Miss\ rate\ or\ false\ negative\ rate\ (FNR) = \frac{FN}{FN+TP}  $$

8 $$ Fall-out\ or\ false\ positive\ rate\ (FPR) = \frac{FP}{FP+TN}  $$

9 $$ F1\ score = \frac{2TP}{2TP+FP+FN}  $$

10 $$ \begin{aligned} Matthews\ correlation\ coefficient\ (MCC) = \\ = \frac{TP\times TN - FP \times FN}{\sqrt{(TP+FP)(TP+FN)(TN+FP)(TN+FN)}} \end{aligned}   $$

where TP is the number of true positives, TN is the number of true negatives, FP is the number of false positive, FN is the number of false negative.

Prediction results are reported in Tables 2 and 3 for cel; in Tables 4 and 5 for hsa, respectively. As for Tables 2 and 4, the first two columns of the tables contain respectively the algorithms we compared and the year of the last update. Third column contains, for each method, the subset of the miRNA-mRNA predicted interactions that have at least one miRNA or mRNA belonging to the validated target dataset. Finally, the last two columns report the number of TP and TN with regards to the total number of positive validated interactions and negative validated interactions.

**Table 2:** Comparison among the proposed method and the other prediction algorithms for the *C. elegans* species, in terms of true positive and true negative interactions

Validation of miRNA target prediction algorithms for *C. elegans*				
Algorithm	Last update (year)	Predicted interactions	3209 positive validated interactions	16 negative validated interactions
			True positive	True negative
PITA	2008	4874	979	14
MiRanda	2010	3307	829	12
MirSOM	2011	1734	588	15
DIANA-microT	2012	1232	172	16
MiRNATIP	2015	6533	994	15

**Table 3:** Performances of prediction algorithms related to validated interactions in Table 2

Algorithm	ACC %	PPV %	TPR %	TNR %	FNR %	FPR %	F1 %	MCC
PITA	30.8	99.8	30.5	87.2	69.5	12.5	46.7	0.02750
MiRanda	26.7	99.5	25.8	75.0	74.1	25.0	41.0	0.00133
MirSOM	18.7	99.8	18.3	93.7	81.6	6.2	30.9	0.02195
DIANA-microT	5.8	100.0	5.3	100.0	94.6	0.0	10.2	0.01676
miRNATIP	31.2	99.9	30.9	93.7	69.0	6.2	47.3	0.03761

Statistical measures reported in this table are accuracy (*ACC*), precision (*PPV*), sensitivity (*TPR*), specificity (*TNR*), miss-rate (*FPR*), F1-measure (*F1*) and Matthews correlation (*MCC*), respectively

**Table 4:** Comparison among the proposed method and the other prediction algorithms for the Homo sapiens species, in terms of true positive and true negative interactions

Validation of miRNA target prediction algorithms for Homo sapiens				
Algorithm	Last update (year)	Predicted interactions	3209 positive validated interactions	16 negative validated interactions
			True positive	True negative
PITA	2008	43823	1971	109
MiRanda	2010	420800	9962	73
TargetScan	2012	105407	4367	96
Pictar	2012	40497	2713	100
DIANA-microT	2012	367379	7805	91
MiRNATIP	2015	968798	11945	86

**Table 5:** Performances of prediction algorithms related to validated interactions in Table 4

Algorithm	ACC %	PPV %	TPR %	TNR %	FNR %	FPR %	F1 %	MCC
PITA	5.3	99.3	5.0	88.6	94.9	11.4	9.6	–0.01617
MiRanda	25.5	99.5	25.4	59.3	74.5	40.6	40.5	–0.01946
TargetScan	11.4	99.3	11.2	78.0	88.8	21.9	20.0	–0.01912
Pictar	7.1	99.1	6.9	81.3	93.1	18.7	12.9	–0.02581
DIANA-microT	20.1	99.5	19.9	74.0	80.0	26.0	33.2	–0.00847
miRNATIP	30.6	99.7	30.5	69.9	69.4	30.1	46.7	0.00055

Statistical measures reported in this table are accuracy (*ACC*), precision (*PPV*), sensitivity (*TPR*), specificity (*TNR*), miss-rate (*FPR*), F1-measure (*F1*) and Matthews correlation (*MCC*), respectively

Tables 3 and 5, for cel and hsa respectively, show the prediction scores computed using all the statistical measures presented in Eqs. 3 to 10.

Observing these results, it is possible to notice that our miRNATIP algorithm reaches the best scores regarding the sensitivity, with a score of almost 31 % for both cel (30.97 %) and hsa (30.54 %) species. At the same time we produced the largest number of total predicted interactions. The other best predictors are PITA for cel (30 %) and MiRanda for hsa (about 25 %). Best results are also reached in terms of accuracy and F1 score, confirming the fact that our algorithm predicts the most number of correct interactions.

As for the specificity scores, our miRNATIP algorithm is able to reach one of the best results especially for cel species. In fact the proposed predictor obtained a score of 93.75 %. The best result in terms of specificity for cel is reached by DIANA-microT tool (100 %), but it produced very low sensitivity score (5.36 %). As regards hsa, mirRNATIP specificity score (69.92 %) is consistent with the scores reached by the other algorithms, whose best specificity score is reached by PITA (88.62 %). Once again, however, PITA reached a very low sensitivity score of about 5 %.

miRNATIP proves to reach the lowest miss-rate (FNR), whereas the fall-out score (FPR) is the second best for cel and and the fifth for hsa. Finally, miRNATIP is the only algorithm producing a positive MCC, confirming the its goodness of the overall predictive power.

It is important to notice that although we predicted the largest number of interactions with respect to the other methods for both species, we obtained a fair specificity score, with regards to the other predictors, and the best sensitivity score. That that means our method could predict more potentially true miRNA-mRNA interactions than the other algorithms.

All the predicted interaction are freely available at the following url: http://tblab.pa.icar.cnr.it/public/miRNATIP/.

## Conclusion and future work

The interaction between miRNA and mRNA is of fundamental importance in the post-transcriptional regulatory mechanism. In this paper we presented miRNATIP, a SOM-based predictor for the identification of miRNA-target interactions. MiRNATIP simulates the main features of the miRNA-mRNA interaction, including near perfect seed pairing, the presence of bulges, free energy constraints for stability of the duplex. In particular a SOM is trained considering only the miRNA seeds (first 8 nucleotides), that are represented by means of a numerical encoding derived from as PWM, and then 8-mer mRNA fragments are projected over the trained lattice in order to identify a preliminary list of putative interactions. Then that list is filtered out taking into account the distance between the remaining parts of the miRNA and mRNA sequences and the free energy values. The obtained predictions, for cel and hsa species, have been validated in terms of sensitivity and specificity scores against six other state-of-the-art predictors (miRanda, PITA, DIANA-microT, mirSOM, PicTar, TargetScan) with regards to a manually curated dataset of both validated miRNA-mRNA interactions and validated non-target interactions. Results demonstrated that our methods reached the best sensitivity score for both species and a specificity score consistent with the other predictors, even if we produced the largest number of putative interactions. As future work, we are going to test our method with other species, and at the same time we will provide a web service that will allow to download already computed predictions or to test our algorithm with customized sets of miRNA and/or mRNA sequences.
